# Biomaterial Scaffolds for Periodontal Tissue Engineering

**DOI:** 10.3390/jfb15080233

**Published:** 2024-08-20

**Authors:** Huanhuan Chen, Guangying Song, Tianmin Xu, Chenda Meng, Yunfan Zhang, Tianyi Xin, Tingting Yu, Yifan Lin, Bing Han

**Affiliations:** 1 Department of Orthodontics, School and Hospital of Stomatology, Peking University, Beijing 100081, China; 1510303108@bjmu.edu.cn (H.C.); songguangying@pkuss.bjmu.edu.cn (G.S.); 9264011531@bjmu.edu.cn (T.X.); 2111210452@bjmu.edu.cn (C.M.); yunfanzhang@bjmu.edu.cn (Y.Z.); xintianyi@bjmu.edu.cn (T.X.); tingtingyu@bjmu.edu.cn (T.Y.); 2 National Engineering Laboratory for Digital and Material Technology of Stomatology, Beijing Key Laboratory of Digital Stomatology, Beijing 100081, China; 3 Division of Paediatric Dentistry and Orthodontics, Faculty of Dentistry, The University of Hong Kong, Hong Kong SAR, China

**Keywords:** biomaterials, periodontal tissue regeneration, periodontitis, scaffolds, tissue engineering

## Abstract

Advanced periodontitis poses a significant threat to oral health, causing extensive damage and loss of both hard and soft periodontal tissues. While traditional therapies such as scaling and root planing can effectively halt the disease’s progression, they often fail to fully restore the original architecture and function of periodontal tissues due to the limited capacity for spontaneous regeneration. To address this challenge, periodontal tissue engineering has emerged as a promising approach. This technology centers on the utilization of biomaterial scaffolds, which function as three-dimensional (3D) templates or frameworks, supporting and guiding the regeneration of periodontal tissues, including the periodontal ligament, cementum, alveolar bone, and gingival tissue. These scaffolds mimic the extracellular matrix (ECM) of native periodontal tissues, aiming to foster cell attachment, proliferation, differentiation, and, ultimately, the formation of new, functional periodontal structures. Despite the inherent challenges associated with preclinical testing, the intensification of research on biomaterial scaffolds, coupled with the continuous advancement of fabrication technology, leads us to anticipate a significant expansion in their application for periodontal tissue regeneration. This review comprehensively covers the recent advancements in biomaterial scaffolds engineered specifically for periodontal tissue regeneration, aiming to provide insights into the current state of the field and potential directions for future research.

## 1. Introduction

Periodontitis is a complex inflammatory disease, influenced by multiple factors, that gradually infiltrates the tooth-supporting apparatus, ultimately leading to gingival recession, loss of periodontal attachment, alveolar bone resorption, tooth mobility, and even tooth loss [1]. This disease entails an intricate and dynamic interplay between distinct bacterial pathogens, detrimental host immune reactions, and external factors such as smoking and diabetes mellitus [2]. Recently, advancements have been made in the classification of periodontal diseases, aiming to better capture the diverse manifestations and underlying mechanisms of this condition. In order to successfully manage periodontitis, a comprehensive understanding of its pathogenesis, primary etiology, risk factors, contributing factors, established treatment protocols, and the new classification system is crucial [3]. For mild to moderate periodontitis, mechanical scaling and root planing, occasionally supplemented with adjuvant therapy involving antiseptics or antibiotics, are adequate measures to eliminate the underlying causes and minimize modifiable risk factors. However, in cases of more severe periodontal defects where both soft and hard tissues have been lost, measures to effectively promote new attachment formation on teeth or to reconstruct periodontal supporting tissues become necessary [4,5].

**Figure 1:**
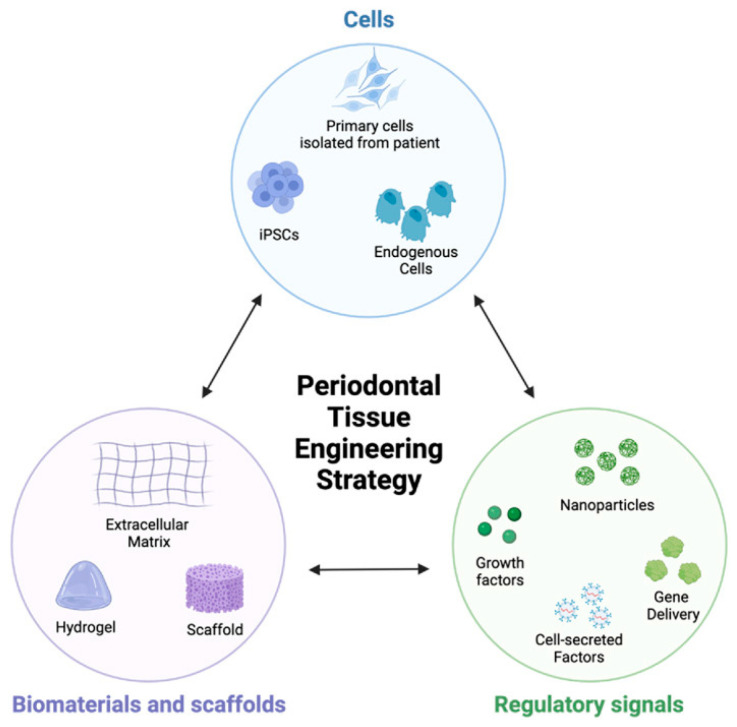
The tissue engineering triad illustrates the three critical design criteria for periodontal tissue engineering strategies. Reprinted with permission from Ref. [15]. 2022, Swanson WB.

Promoting periodontal tissue regeneration and repairing periodontal bone defects remain the ultimate therapeutic objectives in treating periodontal disease [6]. Periodontal tissue engineering, an emerging field within regenerative medicine, focuses on restoring, regenerating, or enhancing periodontal tissues [6,7,8]. Its objective is to reconstruct healthy periodontal ligament (PDL), cementum (CM), alveolar bone, and gingival tissue for patients afflicted with periodontitis or periodontal tissue defects arising from diverse etiologies [9,10,11,12]. Biomaterial scaffolds, bioactive molecules such as growth factors, and stem cells constitute the three pivotal elements in the management of periodontal defects (Figure 1). Biomaterial scaffolds are recognized as a crucial component in tissue engineering due to their significant impact on the biological response of tissue cells and subsequent tissue formation [13,14,15]. Compared to conventional barrier biomaterial approaches, which prevent the downward growth of connective and epithelial tissues into the defective site to favor periodontal tissue regeneration, bioactive scaffolds clearly exhibit enhanced functionality in stimulating the differentiation and osteogenic/cementogenic gene expression of periodontal ligament cells (PDLCs), ultimately leading to improved periodontal regeneration applications [16,17,18]. Additionally, while conventional bone grafting strategies exhibit remarkable osteoconductive, osteoinductive, and osteogenic properties, they have fallen short as preferred alternatives due to various limitations [19]. These include the scarcity of harvestable bone, the necessity of surgical intervention at donor sites, and the potential risks of unforeseen infections, disease transmission, and/or immune rejection. Conversely, periodontal tissue engineering, which leverages the integration of exogenous progenitor cells, biomaterial scaffolds, and bioactive molecules (signals), has emerged as a novel approach to address the intricate architecture and functionality of periodontal tissues, thus opening up a promising new path [20,21,22].

In recent years, remarkable progress has been made in the field of biomaterial science and engineering, leading to extensive research and the exploration of innovative biomaterial scaffolds with favorable biochemical properties for facilitating the regeneration of damaged periodontal tissues [23]. The objectives of this review are to provide a comprehensive overview of the latest advancements in biomaterial scaffolds designed for periodontal tissue regeneration, to delve into discussions regarding their efficacy in both in vitro and in vivo settings, and to explore future perspectives within this field. Additionally, the scope of this review encompasses both natural and synthetic biomaterial scaffolds, with a particular focus on their potential to facilitate the regeneration of damaged periodontal tissues.

## 2. The Role of Biomaterial Scaffolds in Periodontal Tissue Engineering

The periodontal tissue, a vital complex encompassing the periodontal ligament, cementum, alveolar bone, and gingival tissue, is integral to maintaining oral health. This intricate system, however, is vulnerable to chronic and progressive destruction—a process intricately linked to the accumulation of dental biofilm and the resulting dysbiosis mediated by the host immune system. This destructive cascade progresses through distinct stages, manifesting initially as bleeding upon probing, which is a telltale sign of early inflammation. As the disease advances, periodontal attachment loss occurs, with the development of deep probing pockets indicative of the erosion of supporting tissues. Radiographic assessment reveals evidence of alveolar bone resorption—a hallmark of periodontitis. Consequently, teeth may exhibit increased mobility and undergo pathologic migration, further compromising their stability and function.

When addressing periodontitis through conventional, non-surgical, conservative treatment, the aim is to arrest disease progression and promote healing. These interventions often lead to the formation of a long junctional epithelium that attaches to the root surfaces, which represents a biological adaptation, facilitating reattachment and stabilization of the gingival tissues [24,25]. Nevertheless, it should be noted that the protective capability of the long junctional epithelium, which attaches to the root surfaces via hemidesmosomes, is not as effective as the connective tissue fibers anchored in the cementum. The recent advancements in periodontal tissue engineering have significantly reshaped the landscape of treating periodontitis, moving beyond conventional, non-surgical, conservative treatments that often result in the formation of a lengthy junctional epithelium with limited protective capabilities. This evolution has been fueled by the emergence of innovative strategies, particularly guided tissue regeneration (GTR) therapy and the subsequent development of sophisticated biomaterial scaffolds. GTR utilizes the principle of contact inhibition to stimulate the regeneration of periodontal tissue [26,27,28]. However, the regeneration of multiple types of periodontal tissues through GTR are not entirely satisfactory [29]. The occurrence of root resorption and ankylosis is still observable, and the regenerative capacity of the PDL and CM can be significantly compromised when chronic periodontitis recurs. To overcome these limitations, biomaterial scaffolds have garnered immense attention as they offer a tailored platform for delivering bioactive cues that can significantly enhance periodontal tissue regeneration. These scaffolds are not merely passive structures but active participants in the engineering process, playing multifaceted and pivotal roles [30]. Recent developments in biomaterial scaffolds emphasize the integration of advanced materials science, nanotechnology, and biomimicry principles to create scaffolds that mimic the native ECM of periodontal tissues [31]. Here are the key functions of biomaterial scaffolds:

- Provide a three-dimensional structure;
  ▪ Scaffolds serve as a three-dimensional (3D) template that mimics the natural ECM of periodontal tissues [32,33]. This 3D structure provides the necessary environment for cells to adhere, proliferate, and differentiate into the desired tissue type [11].
- Support cell growth and differentiation;
  ▪ The scaffold material should be biocompatible and promote cell growth. It provides a surface for cells to attach and spread, enabling them to proliferate and differentiate into the specific cell types required for periodontal tissue regeneration [29,34,35,36,37].
- Enhance vascularization;
  ▪ Some scaffolds are designed to promote angiogenesis, the formation of new blood vessels, which is crucial for nutrient supply and waste removal during the regeneration process.
- Modulate immune response;
  ▪ Advanced scaffolds can modulate the host immune response, reducing inflammation and promoting a regenerative microenvironment conducive to tissue healing.
- Guide tissue regeneration;
  ▪ The scaffold’s porosity and interconnectivity allow for the ingrowth of blood vessels and other supporting tissues, which are essential for the long-term survival and function of the regenerated tissue. The scaffold’s design can be tailored to guide the growth of new tissue in a specific direction or pattern [38,39].
- Deliver growth factors and other biologics;
  ▪ Scaffolds can be loaded with growth factors, such as fibroblast growth factor (FGF) or bone morphogenetic proteins (BMPs), to stimulate and enhance tissue regeneration. These growth factors are gradually released from the scaffold, providing a sustained stimulus for tissue repair and regeneration [37,40,41].
- Mechanical support;
  ▪ The scaffold provides mechanical support to the regenerating tissue, helping to maintain its shape and integrity during the healing process [34]. This is especially important in periodontal tissue engineering, where the regenerated tissue needs to integrate seamlessly with the surrounding tissues [42].
- Personalization and precision;
  ▪ With the advent of precision medicine, biomaterial scaffolds are being tailored to individual patient needs, incorporating patient-specific cells and factors to enhance the efficacy and predictability of periodontal tissue regeneration.

Among the role of biomaterial scaffolds in periodontal tissue engineering mentioned above, the incorporation of bioactive molecules and stem cells in biomaterial scaffolds stands out as a particularly promising strategy for enhancing tissue regeneration and promoting the formation of functional periodontal tissues [12,40]. Adding bioactive molecules to scaffolds presents several potential advantages, including enhanced safety with reduced risk of tumorigenicity, improved stability and prolonged storage capabilities, decreased immunogenic potential, facile production and isolation processes, ability to cross biological barriers, and targeted delivery of regenerative factors. A summary of bioactive molecules and stem cells incorporated in biomaterial scaffolds are outlined in Table 1.

## 3. The Fabrication Techniques for Biomaterial Scaffolds

In recent years, periodontal tissue engineering has witnessed significant advancements, particularly in the realm of fabricating sophisticated scaffolds that emulate the intricate architecture and functionality of native periodontal tissues. These scaffolds serve as the cornerstone of tissue-engineered constructs, providing not only structural support but also biochemical cues and an optimal microenvironment crucial for cell growth, differentiation, and, ultimately, tissue regeneration. Typically, three-dimensional scaffolds characterized by high porosity and interconnectivity are preferred for achieving structural and functional recuperation [43,44]. This is because their unique architecture fosters a conducive microenvironment for cell-to-cell communication and seamless scaffold-to-tissue assimilation at the implantation site [45]. During the initial implantation stages, the porous frameworks facilitate blood infiltration into the scaffolds and stabilize blood clots. This process is deemed crucial in initiating tissue restoration and regeneration via enriched vascularization [46]. Notably, macropores in the 100 to 700 μm range promote vascularization at the implanted locations, whereas micropores smaller than 100 μm may hinder cell proliferation due to local ischemia. Additionally, high porosity aids in the diffusion of nutrients, gases, and waste removal, thereby enhancing cellular metabolism and proliferation.

Biomaterials need to be fabricated into suitable scaffolds to perform their functions, and numerous fabrication techniques have been employed to craft these highly porous scaffolds, such as decellularization, salt leaching, electrospinning, and 3D printing, aiming to mimic the intricate structure and function of periodontal tissues [9,10,47,48]. Among them, 3D printing, particularly with bio-inks containing living cells and bioactive molecules, has emerged as a game-changer, enabling the precise control of pore size, permeability, porosity, and even the incorporation of growth factors and signaling molecules. Recent developments in 3D-printing technologies, such as bio-ink optimization, multi-material printing, and the integration of advanced imaging technologies for patient-specific scaffold design, have further enhanced the capabilities of fabricated scaffolds. These advancements allow for the creation of scaffolds with tailored mechanical properties, tailored degradation rates, and the ability to release therapeutic agents in a controlled manner, thereby optimizing the regenerative outcomes. Nonetheless, replicating the intricate complexity of these tissues remains a formidable challenge, which requires scaffolds that not only offer structural integrity but also promote cell growth, differentiation, and tissue regeneration [49,50]. Moreover, the integration of stem cells, particularly mesenchymal stem cells (MSCs) and periodontal ligament stem cells (PDLSCs), within these scaffolds has shown immense promise in periodontal tissue engineering. The combination of these cells with advanced scaffold designs fosters a robust regenerative response, leading to the formation of functional periodontal tissues with improved attachment, bone regeneration, and periodontal ligament reconstruction. The fabrication techniques for biomaterial scaffolds are diverse and depend on the specific requirements of the scaffold, such as pore size, permeability, porosity, and mechanical properties. The common fabrication techniques employed in tissue engineering are outlined in Table 2.

**Table 2:** The common fabrication techniques employed in tissue engineering.

Fabrication Techniques	Mechanisms and Applications	References
Decellularization	The primary goal of decellularization is to eliminate cells and potential antigens from the tissue while preserving the ECM components, which helps reduce the risk of immune rejection when the scaffold is implanted into a host. The decellularization methods include the use of chemical agents and supercritical carbon dioxide.	Liang et al. [32], 2023
Solvent casting and particle leaching	In this method, a polymer solution is mixed with a sacrificial material (e.g., salt particles) that can be later dissolved to create pores. The mixture is cast into a mold, solidified, and then the sacrificial material is leached out, leaving a porous scaffold.	Brown et al. [28], 2015
Gas foaming	This process involves the use of a blowing agent that releases gas during the processing of the polymer, creating pores within the material.	Loh et al. [33], 2013
Phase separation	By manipulating the solubility and phase behavior of polymer solutions, pores can be induced within the scaffold material. This often involves controlling temperature or solvent evaporation to induce phase separation.	Zielińska et al. [30], 2023
Electrospinning	This advanced nanofiber fabrication technique uses an electrostatic field to produce fine polymer fibers, resulting in a highly porous scaffold with nanofibrous structure.	Wang et al. [35], 2023
3D bioprinting	A type of additive manufacturing where cell-laden hydrogels or other biomaterials are precisely deposited layer by layer to form complex 3D structures (Figure 2). This method allows for high precision in scaffold design and can incorporate cells directly into the printed structure.	Miao et al. [40], 2023
Freeze-drying	This technique involves freezing a polymer solution and then sublimating the solvent under reduced pressure, leaving a porous scaffold.	Liang et al. [42], 2023
Rapid prototyping	Methods like selective laser sintering (SLS) or fused deposition modeling (FDM) can be used to create scaffolds with specific designs and porosity.	He et al. [36], 2010
Emulsion templating	This involves the creation of an emulsion (e.g., water in oil) that, upon solidification, leaves pores corresponding to the dispersed phase.	Aldemir et al. [39], 2019

**Figure 2:**
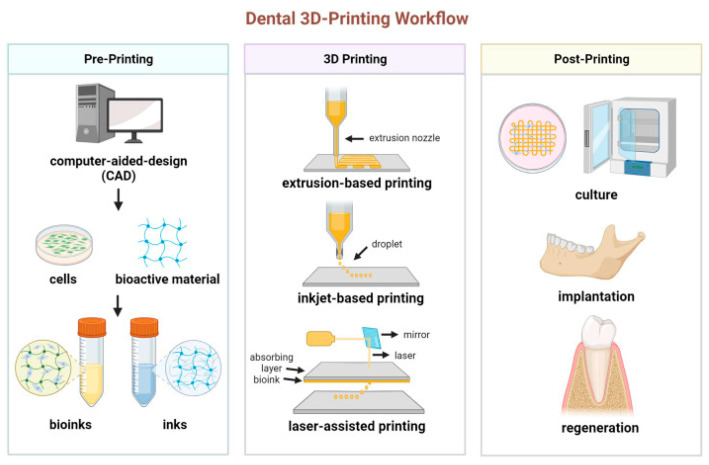
Schematic diagram of the process for 3D printing dental tissue. Reprinted with permission from Ref. [41]. 2024, Zhao F. Extrusion-based printing: using mechanical force or pressure, biomaterials are extruded or dispensed through a nozzle, where the extruded material is then precisely deposited onto a substrate to create the desired 3D structure layer by layer; Inkjet-based printing: the biomaterials are formulated into “bio-inks” that can be deposited in a controlled manner using inkjet-printing technology. The bio-inks are ejected through a printhead with tiny nozzles, allowing for high-resolution patterning of cells and biomaterials; Laser-assisted printing: using a focused laser beam to guide and deposit biomaterials onto a substrate. The laser beam creates a localized energy pulse that induces the transfer of biomaterial from a donor slide to a receiver slide.

## 4. Biomaterial Scaffolds Used for Periodontal Tissue Engineering

Common biomaterial scaffolds used for periodontal tissue engineering include natural polymers such as collagen, chitosan, and hyaluronic acid, as well as synthetic polymers like poly(lactic-co-glycolic acid) (PLGA) and polyethylene terephthalate (PET). These biomaterials have undergone significant modifications to better support periodontal tissue regeneration. For instance, novel formulations and surface modifications have been introduced to PLGA and PET scaffolds, enhancing their biodegradability, cell adhesion properties, and the controlled release of growth factors. This allows for more precise control over the regenerative process, fostering optimal cell growth, differentiation, and tissue remodeling. Additionally, these scaffolds can be fabricated into various forms, including fibers, films, sponges, hydrogels, and porous scaffolds, depending on the specific application and desired tissue regeneration outcomes.

Biomaterial scaffolds can also be classified into monolithic and multiphasic scaffolds. Monolithic scaffolds are composed of a single material or phase, designed to provide a uniform environment for cell growth and tissue formation, whereas multiphasic scaffolds are composed of multiple materials or phases, each with distinct properties, designed to mimic the complex composition and structure of natural tissues.

**Figure 3:**
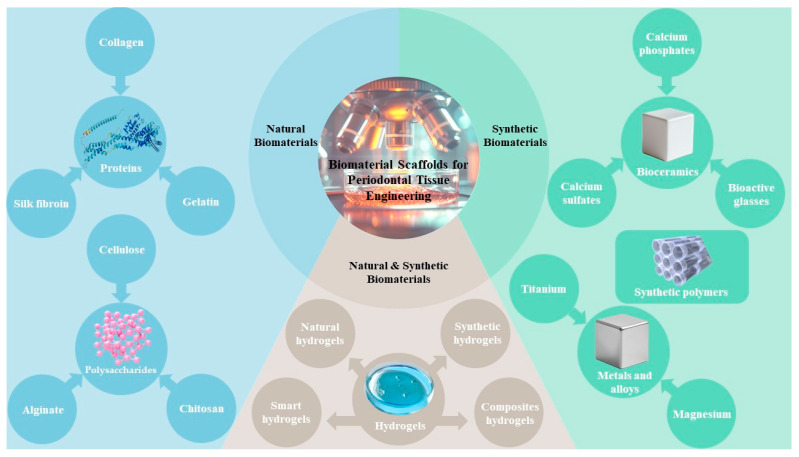
A graphical abstract outlining the biomaterial scaffolds reviewed in this article.

Given that hydrogels encompass both natural and synthetic varieties, apart from detailing natural biomaterials and synthetic biomaterials individually, this review inclusively categorizes hydrogels as a distinct category under natural and synthetic biomaterials. The biomaterial scaffolds that have been recently tested for periodontal tissue regeneration are outlined in Table 3 (Figure 3).

### 4.1. Natural Biomaterials

In periodontal tissue engineering, natural biomaterials are widely used due to their excellent biocompatibility and biodegradability. These materials possess a minimized toxicity profile and rarely elicit inflammatory responses or immune reactions, making them a favorable choice for regenerative periodontal therapies. Natural polymers, exemplified by proteins (such as collagen, silk fibroin, and gelatin) and polysaccharides (such as cellulose, alginate, and chitosan), are commonly regarded as the pioneer biodegradable biomaterials deployed in clinical settings [51]. These polymers possess inherent bioactive qualities, allowing them to engage actively with cellular components. However, natural polymers often exhibit a lack of mechanical stability, and their mechanical and biological attributes can vary considerably based on extraction techniques. Additionally, their heightened sensitivity to enzymatic degradation may lead to inconsistent scaffold resorption and tissue remodeling. Therefore, reinforcement using resilient materials like fibers or hydroxyapatites is frequently considered.

#### 4.1.1. Collagen

Collagen, a protein abundantly present in human skin, bones, blood vessels, and various tissues, exhibits remarkable properties. It possesses low immunogenicity, attracting and activating gingival fibroblast cells while exhibiting hemostatic capabilities [8]. Its superior biocompatibility and biodegradability allow for excellent recognition and adherence by cells [14]. Imber et al. have immunohistochemically evaluated the impact of a volume-stable collagen scaffold (VCMX) on periodontal regeneration [52]. Their findings revealed a favorable effect of VCMXs on angiogenesis, along with a similarly high cell turnover in both the regenerated and original PDL, which supported periodontal regeneration in intrabony defects. Collagen scaffolds have demonstrated the ability to stimulate fibroblast DNA synthesis, and osteoblasts display higher adherence to collagen membrane surfaces compared to alternative surfaces [53]. However, this material has a significant drawback: its rapid fragmentation and degradation upon gingival dehiscence, which can result in membrane exposure and subsequently hinder bone regeneration. To address these limitations, various strategies have been explored, including physical, chemical, and enzymatic managements, as well as crosslinking methods like ultraviolet (UV) radiation, genipin (Gp), and glutaraldehyde managements, all aiming to bolster the durability and mechanical strength of collagen scaffolds. Moreover, to enhance periodontal tissue regeneration, the collagen scaffolds are often adorned with additional bioactive cues. For example, Kanemoto et al. pioneered a plasma- and precursor-guided biomimetic method, which enables the coating of a porous collagen scaffold with low-crystalline apatite [54]. This innovative approach yielded a scaffold that displayed notably improved characteristics, including water absorption, calcium release, lysozyme adsorption, and collagenase resistance, compared to its uncoated counterpart. Additionally, the apatite-coated scaffold effectively fostered the osteoblastic differentiation of MC3T3-E1 cells while simultaneously inhibiting their proliferation.

#### 4.1.2. Silk Fibroin (SF)

SF, also known as silk protein, is a natural macromolecular fibrous protein renowned for its exceptional mechanical properties, biocompatibility, biodegradability, proteolytic degradability, minimal inflammatory reaction, and cost-effective processing [55]. By incorporating cells, growth factors, or other bioactive agents into SF scaffolds, researchers aim to mimic the natural ECM environment of periodontal tissues, thus promoting regeneration [56]. Nanofibers produced through electrospinning have garnered significant interest in tissue engineering because of their exceptional mechanical properties, high specific surface area, and resemblance to the ECM morphology [57]. Nevertheless, to achieve successful periodontal regeneration with silk fibroin, several challenges must be tackled, including ensuring an optimal scaffold structure, regulating the degradation rate, and attaining the desired cell infiltration and tissue integration. Geão et al. created a SF solution blended with glycerol (GLY) or polyvinyl alcohol (PVA) at various weight ratios, up to 30%. These blended membranes showed remarkable ductility, crucial for easy handling and adapting to defects. After culturing with human periodontal ligament fibroblast cells (hPDLs) for seven days, the membranes demonstrated enhanced hydrophilicity and proteolytic degradability compared to pure silk fibroin membranes. Remarkably, SF/GLY blends stand out in terms of cell adhesion and viability, successfully preserving the proper morphology of hPDLs, thus emerging as promising candidates for periodontal regeneration applications [58]. Serôdio et al. innovatively designed a silk fibroin (SF)/poly(ethylene oxide) (PEO) membrane. This membrane was crafted by integrating ultrasound sonication before the electrospinning process (ranging from 0 to 20 min). Their strategy was focused on physically adjusting the rheological properties of the solutions (consisting of 10 to 30% *w*/*v* PEO) to optimize its spinnability. The rheological assessments revealed that sonication significantly boosts the viscosity of SF/PEO solutions, thereby elevating the quality of the resulting electrospun fibers and consequently improving their mechanical properties, both in dry and wet environments. Additionally, this approach positively influenced the in vitro cellular behavior of hPDLs, leading to a notable surge in cell proliferation [59].

#### 4.1.3. Gelatin

Gelatin is one of the promising biomaterials with excellent cytocompatibility, hydrophilicity, adequate degradability, and water solubility [60]. Derived from collagen, gelatin can be produced through acid or alkaline hydrolysis [61]. Interestingly, gelatin exhibits contrasting gelation behavior to collagen; it forms gels at temperatures below 25 °C, whereas it transitions into a liquid state at physiological temperature (37 °C). This sol–gel shift occurs at approximately 30 °C. By incorporating chemicals like genipin or modifying the gelatin structure with components like methacrylate, the stability of its 3D form can be enhanced under physiological conditions. Furthermore, gelatin’s Arg-Gly-Asp sequences are pivotal for cell proliferation, and its ability to copolymerize with alginate leverages the favorable traits of both materials, which is beneficial for osteogenic differentiation of adipose-derived stem cells in 3D cultures. Xu et al. created a three-dimensional (3D) multilayered scaffold through the stacking and stabilization of electrospun polycaprolactone/gelatin (PCL/Gel) fibrous membranes [62]. This innovative biomaterial exhibited impressive hydrophilic and mechanical properties, effectively supporting the adhesion and proliferation of hPDLSCs. In a rat model simulating acute periodontal defects, the scaffold demonstrated its ability to guide cellular orientation, promote further differentiation, and facilitate new bone regeneration, as well as angular concentrated fiber regeneration on the root surface of the defect. These regenerative effects closely resembled those observed in normal periodontal tissue. Cho et al. fabricated Poly(L-lactic acid) (PLLA) and PLLA/gelatin polymer membranes through electrospinning to assess how the content of PLLA and gelatin influenced various properties of the membranes, including mechanical strength, water uptake capacity (WUC), water contact angle (WCA), degradation rate, cytotoxicity, and cell proliferation. The findings indicate that the adjustable PLLA/gelatin hybrid membrane is highly suitable for absorbable GTR. Nevertheless, further research is required to conduct in vivo testing on the combination of bone grafts and hydrophilic barrier membranes enriched with growth factors, antibiotics, and regenerative cells for a comprehensive clinical evaluation [63].

#### 4.1.4. Cellulose

Cellulose, an extracellular polysaccharide excreted by bacteria, stands out as a remarkable natural polymer due to its fascinating attributes, such as biocompatibility, high water retention capacity, high purity and crystallinity, good chemical stability, low cost, and ease of processing. Bacterial cellulose (BC)-based compounds, such as BC/collagen, BC/gelatin, BC/fibroin, and BC/chitosan, offer enhanced properties and functionality, enabling a wide range of biomedical applications. BC-based magnetic nanocomposites offer extra magnetic functionality, enhancing the already impressive characteristics of pure BC. This combination renders them highly suitable potential materials for a range of medical and environmental applications [64]. BC has also been used to devise a customizable matrix that enables the synthesis of various kinds of calcium carbonate (CaCO_3_) and hydroxyapatite crystals from diverse starting reagents, thereby enhancing biocompatibility [65]. Chiaoprakobkij et al. discovered that curcumin-loaded BC, produced via a straightforward, practical, and economical mechanical blending and casting process, possessed significant antibacterial properties against *Escherichia coli* (*E. coli*) and *S. aureus*. Additionally, it demonstrated non-cytotoxicity towards human keratinocytes and human gingival fibroblasts, while displaying strong anticancer effects in oral cancer cells [66]. An et al. have developed a resorbable BC membrane using electron beam irradiation for guided bone regeneration (GBR). Comprehensive mechanical, chemical, and biological evaluations of these electron beam-irradiated BC membranes (EI-BCMs) showed effective cellular interaction and bone regeneration facilitation [67]. Peng et al. produced a magnesium oxide (MgO) nanoparticles-incorporated PCL/gelatin core-shell nanocellulose (Coaxial-MgO) periodontal membrane fabricated by way of a coaxial electrospinning technique. It was found that the integration of MgO nanoparticles had minimal impact on the morphology and mechanical properties of the nanocellulose membranes. The coaxial-MgO, featuring a core-shell fiber structure, exhibited superior hydrophilic properties, a steady release of magnesium ions (Mg^2+^), and enhanced proliferation rates and adhesion of hPDLSCs. Additionally, it demonstrated excellent biocompatibility, hydrophilicity, elevated alkaline phosphatase (ALP) activity, the formation of mineralized nodules, upregulation of osteogenic-related genes, and potent antibacterial properties against *E. coli* and Actinobacillus actinomycetemcomitans (A. a) [68].

#### 4.1.5. Alginate

Alginate is a non-toxic, biocompatible, and biodegradable natural polysaccharide that gels under mild conditions. Additionally, it serves to improve the compatibility, binding, homogeneity, and dispersibility of polymer blends. Despite lacking inherent binding sites for mammalian cell attachment, alginate can be modified by incorporating the adhesion ligand arginine-glycine-aspartic acid (RGD), which facilitates cell attachment. Alginate stands out as an exceptional biomaterial, capable of imparting distinct cellular interactive traits that permit precise control over the long-term gene expression of cells encapsulated within the hydrogel [69]. Zhang et al. developed a bi-crosslinking viscoelastic hydrogel by integrating phenylboronic acid-modified alginate with spermidine (Alg-PBA/Spd). When the hydrogel degrades, spermidine, an anti-inflammatory agent, is gradually released. This Alg-PBA/Spd hydrogel exhibits biocompatibility, injectability, and the ability to rapidly conform to intricate periodontal structures owing to its dynamic crosslinking. Studies conducted on rat models revealed that this viscoelastic hydrogel significantly boosts the deposition of periodontal collagen and hastens the healing of periodontal damage [70]. Qiu et al. innovated a xeno-free alginate-fibrin-platelet lysate (Alg + Fib + hPL) hydrogel incorporated with hPDLSCs for promoting dental regeneration. This innovative hydrogel significantly enhanced the survival and proliferation of hPDLSCs during culturing in a hPL-based medium. Additionally, the encapsulated hPDLSCs, upon exposure to an osteogenic medium containing hPL, demonstrated effective differentiation into the osteoblast lineage. This differentiation was marked by significantly increased expressions of ALP, Rux-2, collagen I, and Osteogenicpontin, ranging from 3 to 10 times their original levels. These findings suggest that the delivery of hPDLSCs via Alg + Fib + hPL holds significant promise for periodontal regeneration and oral and maxillofacial reconstruction, as well as the repair of various bone defects [71].

#### 4.1.6. Chitosan

Chitosan, a natural anionic polysaccharide derived from deacetylated chitin, boasts a flexible structure and an ample number of functional groups. It is extracted from the exoskeletons of crustaceans, fungi, and insects [72]. Chitosan stands out as a remarkable biomaterial, possessing excellent attributes like biodegradability, biocompatibility, antimicrobial properties, antioxidant activity, hygroscopicity, low antigenicity, and exceptional safety [73]. Chitosan consists of a linear polymer chain composed of glucosamine and N-acetyl glucosamine units, connected by β-1,4-glycosidic bonds, featuring free amino groups that enhance its reactivity and solubility compared to chitin. This unique composition allows chitosan to form hydrogels, thanks to the abundance of hydroxyl and/or amine groups available for various chemical reactions [74]. Xu et al. employed chitosan (CS), β-sodium glycerophosphate (β-GP), and gelatin to fabricate an injectable, thermosensitive hydrogel that can steadily dispense aspirin and erythropoietin (EPO). These compounds respectively exhibit anti-inflammatory and tissue regenerative pharmacological actions. The results indicate that CS/β-GP/gelatin hydrogels can be readily formulated as drug delivery vehicles, boasting exceptional biocompatibility. Additionally, aspirin and EPO-loaded CS/β-GP/gelatin hydrogels display notable performance in reducing inflammation and promoting periodontium regeneration, positioning them as a potential therapeutic option for periodontitis in dental practices [75]. Tan et al. have also explored the potential of an injectable, thermosensitive hydrogel composed of β-tricalcium phosphate (β-TCP) and chitosan for carrying cells and promoting periodontal tissue regeneration. This novel biomaterial demonstrated thermosensitive capabilities along with outstanding physical, chemical, and biological characteristics. Furthermore, the incorporation of β-TCP significantly improved the structure and properties of the hydrogels. This preliminary research indicates considerable promise for the use of β-TCP-loaded thermosensitive chitosan hydrogels as scaffolds in periodontal bone and soft tissue repair procedures [76]. Amir et al. explored the feasibility of utilizing PDL cell sheets along with arginine-glycyl-aspartic acid (RGD)-modified chitosan scaffolds for periodontal tissue regeneration. Clinical assessments indicated that the group treated with PDL cell sheets and RGD-modified chitosan scaffolds demonstrated increased epithelial attachment formation, higher alveolar bone density around the transplanted region, elevated CEMP-1 protein expression, and greater periodontal tissue formation [77].

### 4.2. Synthetic Biomaterials

Synthetic biomaterials, consisting of bioceramics, synthetic polymers, metals, and alloys, have found widespread use in various clinical applications due to their remarkable mechanical properties and formability. These materials, engineered to mimic natural biological tissues, offer unique advantages in medical procedures, such as durability, biocompatibility, and the ability to be shaped and molded to meet specific surgical needs. Their versatility and performance have made them indispensable tools in periodontal regeneration [78].

#### 4.2.1. Bioceramics

Bioceramics, ceramic-based biomaterials renowned for their exceptional biocompatibility and distinctive physical properties, are extensively employed in medical applications, particularly in direct contact with living tissue [79]. These materials are categorized into bioinert and bioactive ceramics. Bioinert ceramics, known for their chemical stability and excellent wear resistance, primarily serve as structural implants, such as bone screws and hip joints. However, they also find application in periodontal regeneration where stability and non-reactivity are paramount. On the other hand, bioactive ceramics establish a chemical bond with adjacent bone tissue, fostering osteointegration. Calcium phosphates materials like hydroxyapatite (HA) and β-tricalcium phosphate (β-TCP) are frequently utilized in periodontal regeneration due to their ability to stimulate tissue growth and integration [80]. These bioceramics possess notable strengths in biocompatibility and osteoconductivity, yet they are challenged by their slow bioresorbability. Once implanted, sintered HA is rarely resorbed and persists in the body for extended periods, thus limiting its standalone application. Consequently, there has been a surge of interest in β-TCP in recent years. β-TCP demonstrates degradation kinetics that align with the rate of new bone growth and possesses regenerative capabilities akin to autologous bone grafts. However, β-TCP falls short in terms of mechanical properties, and in cases of significant bone defects, its resorption rate may be excessively rapid. Therefore, achieving a harmonious balance between HA and β-TCP is pivotal for attaining the desired mechanical strength, appropriate degradation kinetics, and osteointegration within biphasic calcium-phosphate ceramics. Numerous studies have been conducted to ascertain the optimal HA/β-TCP ratio but drawing robust comparisons among the results is challenging due to the influence of various factors on the research outcomes across different groups [81,82,83].

Besides calcium phosphates materials, calcium sulfate (CS), popularly referred to as plaster of Paris, holds significant importance in periodontal regeneration. It is frequently employed as an exemplary bone graft material or as a constituent of bone graft substitutes due to its biodegradability, bioresorbability, and osteogenic traits. During periodontal regeneration surgeries, calcium sulfate facilitates bone growth and healing by providing a scaffold for osteoblast adhesion and proliferation. Nevertheless, its limited solubility control and unpredictable behavior when exposed to blood and saliva have gradually decreased its application over the past years. To address the issues related to rapid resorption, calcium sulfate has been combined with other materials, including calcium phosphates, to achieve a more stable structure and finer resorption kinetics control. Li et al. formulated a calcium phosphate/calcium sulfate cement reinforced with sodium carboxymethyl cellulose (CMC/OPC), which exhibited suitable physicochemical properties for clinical use [84]. Xu et al. manipulated the solidification of CS by incorporating β-sheet-rich silk fibroin nanofibers (SFF), striving for superior mechanical and biological attributes. The SFF-induced CS exhibited a reduced size, an increased number of filament structures, and attained enhanced mechanical and biological qualities [85].

Apart from the bioceramics mentioned above, bioactive glasses (BGs) have gained considerable attention owing to their bioactive characteristics and remarkable ability to deliver therapeutics in tissue engineering applications [86]. Recent studies have uncovered that bioactive glasses exhibit osteoinductive properties. These materials possess the capability to induce osteoprogenitor cells to migrate into the graft structure and exert an influence on the gene expression of undifferentiated cells, ultimately promoting cell differentiation [87]. The release of therapeutic ionic species, particularly silicate and calcium ions, is attributed to these exceptional qualities, efficiently prompting bone cells towards paths of regeneration and self-repair [88]. Additionally, specific types of glasses, such as mesoporous bioactive glasses (MBGs), serve as optimal delivery platforms for diverse small molecules and pharmaceutical agents. According to Balasubramanian et al., incorporating boron into bioactive glasses in various proportions significantly impacts the glass structure, processing parameters, biocompatibility, biodegradability, bioactivity, and cytotoxicity [89]. Zambon et al. reviewed the bioactivity, cytocompatibility, antibacterial, and antioxidant properties, as well as osteogenic and angiogenic capabilities, of cerium-doped bioactive glasses (Ce-BGs), proposing their potential application in the reconstruction of both hard and soft tissues [90].

#### 4.2.2. Synthetic Polymers

Synthetic polymers are ideal biodegradable scaffold materials in tissue engineering, owing to their mechanical robustness and excellent biocompatibility [63]. By adjusting their molecular weight and chemical composition, these scaffolds can be customized to exhibit the desired biodegradability and mechanical properties. However, contrary to the hydrophilicity of natural polymers, synthetic polymers are biologically inert, and their hydrophobic properties may prevent blood infiltration, affecting scaffold integration at the implanted site. To address this issue, techniques like plasma surface activation, as well as the coating or incorporation of bioactive molecules, are often used [91,92]. The chemical structure of polymeric scaffolds plays a pivotal role in determining a wide array of properties. Surface properties, in particular, exert a significant influence as they shape the polymers’ interactions with cells and proteins. Key surface properties, such as wettability, swelling ability, electrostatic effects, hydrolytic degradation, elasticity, and morphology, collectively impact the adjacent interfacial environment, thereby facilitating protein interactions on the scaffold’s surface [93]. Then, plasma surface activation enhances wettability and biocompatibility, which are crucial for GTR. Coatings or bioactive molecule additions encompass ECM proteins, growth factors, and specialized proresolving mediators, along with diverse antibiotics and anti-inflammatory medications. Generally, modifying synthetic polymers in this way does not significantly change their bulk properties but improves the material’s interaction with surrounding tissues [94]. Malekpour et al. used the electrospinning method to produce simvastatin-loaded poly(lactic-co-glycolic acid) nanofibers (SIM-PLGA-NF). After optimizing these nanofibers (with a diameter of 640.2 ± 32.5 nm and a simvastatin entrapment efficiency of 99.6 ± 1.5%), they enhanced their surface with a 1% *w*/*v* hyaluronic acid solution, creating 1%HA-SIM-PLGA-NF. This modification aimed to improve their compatibility with fibroblasts and make them a more viable scaffold option for periodontal tissue engineering. Clinical assessments showed that the altered nanofibers displayed a consistent, bead-free, intertwined structure reminiscent of the ECM. Additionally, they demonstrated excellent mechanical properties and positively impacted the proliferation, adhesion, and differentiation of fibroblast cells [95]. Zhang et al. employed an innovative Janus porous polylactic acid membrane (PLAM) fabricated by merging unidirectional evaporation-induced pore formation with the self-assembly of a bioactive metal-phenolic network (MPN) nanointerface. The resulting PLAM-MPN membrane uniquely features a barrier function on its dense side and a bone-forming capability on its porous side. When implanted into rat periodontal bone defects, PLAM-MPN significantly promoted bone regeneration, indicating its diverse abilities to modulate cell physiology in favor of bone regeneration [96]. Zhang et al. utilized side-by-side electrospinning to produce nanofibrous membranes made up of polylactic acid (PLA) and polycaprolactone (PCL) fibers. To enhance their hydrophilic characteristics, various concentrations of gelatin were incorporated into the fiber membranes. The findings reveal that PCL/PLA dual-fibrous composite membranes containing 30% gelatin exhibit adequate mechanical strength, along with improved hydrophilic properties [97].

#### 4.2.3. Metals and Alloys

Metals and alloys are selectively used to facilitate periodontal tissue engineering. Commonly, titanium and titanium alloys (nickel–titanium) are widely used in periodontal regeneration due to their excellent biocompatibility, corrosion resistance, and mechanical properties. The surface of titanium can be crafted with a mesh structure, which enhances its porosity. This, in turn, boosts cell proliferation and mesenchymal stem cell differentiation, ultimately promoting osseointegration. Urban et al. used a titanium-reinforced polytetrafluoroethylene (PTFE) mesh for vertical bone augmentation (VBA) in alveolar ridges with deficiencies. They discovered that combining the titanium-reinforced PTFE mesh with autologous bone and xenograft can be a safe and reliable method for achieving bone growth [98]. Lee et al. assessed the complication rates and vertical bone gain (VBG) following GBR by comparing two distinct materials: dense PTFE titanium-reinforced membranes versus titanium mesh draped with cross-linked collagen membranes. Their findings revealed that both GBR methods produced comparable results in terms of complications, vertical bone gain, and implant stability when used for the restoration of the atrophic posterior mandible [99]. Recent studies have demonstrated the beneficial effects of periodontal scaffolds fabricated from a combination of collagen and TiO_2_ nanoparticles, with the aim of promoting a harmonious interaction between bodily tissues and biomaterials. Pullisaar et al. crafted porous titanium dioxide (TiO_2_) scaffolds layered with alginate hydrogel enriched with simvastatin [100]. The integration of TiO_2_ scaffolds’ physical traits with simvastatin’s osteogenic properties offers a fresh approach for bone regeneration, especially in situations where immediate load-bearing capacity is essential or unfeasible. Elango et al. developed a cutting-edge 3D matrix, utilizing collagen blended with sodium alginate and titanium oxide (TiO_2_) [101]. This matrix recreates the in vivo microenvironment, serving as a foundation for culturing human periodontal ligament fibroblasts (HPLFs) towards osteogenic differentiation. This innovative 3D structure encourages HPLF cells to differentiate into osteoblastogenic lineage cells in vitro, marking it as a promising avenue for further exploration in treating periodontal tissue damage in vivo.

In addition to titanium and titanium alloys, magnesium (Mg) has emerged as a biodegradable material that is increasingly drawing attention, thanks to its beneficial mechanical properties and remarkable biocompatibility. Magnesium frequently finds use in combination with other substances to craft composites or serve as a dopant, thereby augmenting the characteristics of scaffolds or biomaterials. By integrating magnesium into bioactive composites or scaffolds, we can bolster the biocompatibility and bioactivity of these materials, thus stimulating the growth of periodontal tissue and revitalizing periodontal structures. Furthermore, magnesium can be employed as a dopant in materials like hydroxyapatite, adjusting its properties to more closely resemble the natural bone setting and potentially elevating its osteogenic potential. Shoba et al. developed a 3D nano bilayered scaffold that is spatially and functionally graded, impregnated with bromelain-conjugated magnesium-doped hydroxyapatite nanoparticles, and aimed at periodontal regeneration. By functionalizing the polymeric scaffold with bromelain and magnesium-doped hydroxyapatite nanoparticles, the scaffold’s mechanical, physicochemical, thermal, and biological properties were significantly improved. This innovation mimics the complex structure of the ECM, providing essential bioactive cues that regulate cellular functions. The scaffold also demonstrates antibacterial potential, hemocompatibility, and enhances cell proliferation and migration rates in vitro [102]. Additionally, magnesium-based materials can be fashioned to gradually dispense magnesium ions, exerting a favorable influence on the regeneration process. Multiple studies have presented evidence indicating that the integration of MgO nanoparticles into any given substrate significantly enhances osteogenic potential, manifesting in elevated alkaline phosphatase levels, increased bone volume fraction, and greater bone mineral density. The optimal concentration of MgO nanoparticles could serve as a perfect additive to diverse substrates, thereby facilitating bone regeneration [103]. Peng et al. utilized the coaxial electrospinning technique to fabricate MgO nanoparticle-incorporated PCL/gelatin core-shell nanocellulose periodontal membranes (coaxial-MgO). These membranes, characterized by a core-shell fiber structure, demonstrate excellent hydrophilic properties and provide a sustained release of magnesium ions (Mg^2+^). This membrane also demonstrates improved proliferation rates of hPDLSCs, enhanced hPDLSC adhesion, increased alkaline phosphatase (ALP) activity, formation of mineralized nodules, upregulation of osteogenic-related genes, and strong antibacterial properties against *E. coli* and Actinobacillus actinomycetemcomitans (A. a) [68]. The magnesium membrane offers numerous advantages, including mechanical stability, a pliable structure, and a surface conducive to the adhesion and migration of human gingival fibroblasts, while also being fully resorbable. Both in vitro and in vivo testing have demonstrated that fibroblastic cells adhere to and migrate across the degrading surface of magnesium membranes [104]. Hangyasi et al. employed a magnesium membrane (NOVAMag^®^ membrane from botiss biomaterials GmbH, Zossen, Germany) for the treatment of intrabony defects (IBDs). The utilization of magnesium membranes in various shapes for IBD treatment yielded satisfactory functional and esthetic results [105].

### 4.3. Natural and Synthetic Biomaterials

**Figure 4:**
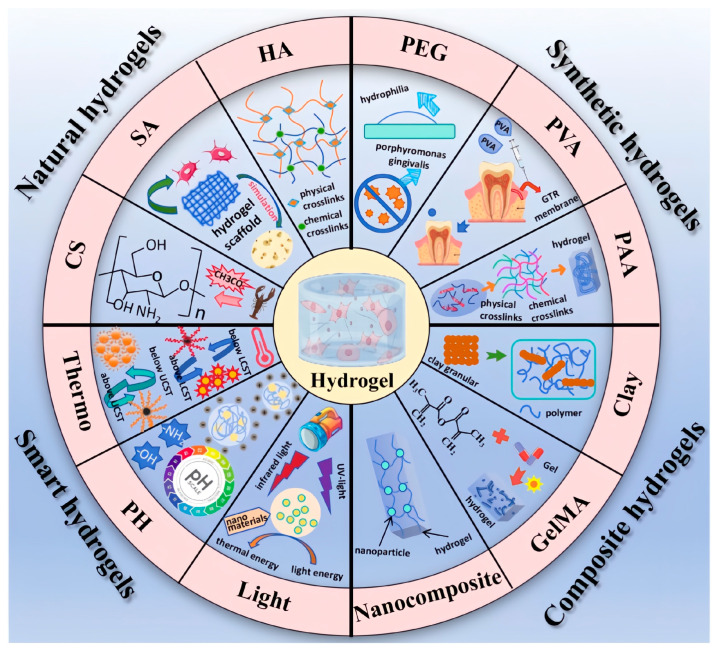
The main classifications of hydrogel. Reprinted with permission from Ref. [106]. 2024, Guo W.

Hydrogels, which could be either natural or synthetic, constitute a category of crosslinked macromolecular polymer networks renowned for their absorption capabilities and hydrophilic traits [106,107], and various types of biomaterials can be formed as hydrogels (Figure 4). The primary components of natural hydrogels are proteins and polysaccharides, preferred due to their natural origin [108], while synthetic hydrogels are formed via physical or chemical crosslinking, resulting in stronger mechanical properties and consistent mass stability. However, natural hydrogels’ clinical applicability is limited by their low mechanical strength and stability, despite demonstrating biocompatible, bioactive, and biodegradable effects in both animal and clinical studies. Conversely, synthetic hydrogels may cause inflammation or cytotoxicity due to the presence of unreacted monomers, initiators, or crosslinkers [109]. To address these issues, various techniques should be employed to craft hydrogels customized for specific applications.

The advantages of hydrogels encompass their remarkable water absorption capacity, excellent biocompatibility, structural design versatility, softness, resistance to deformation, and efficient drug utilization, as well as their convenience and safety [110]. Hydrogels are favored in periodontal regeneration therapy owing to their versatility as barrier membranes, scaffolds, carriers for cell transplantation, and drug delivery systems [111]. By modifying their functional components, the physicochemical characteristics of hydrogels can be customized to align with the distinct mechanical and biological needs of different tissues [112]. For example, Kishen et al. improved the biomechanical properties of hydrogels by adding inorganic fillers. The resulting Hydroxyapatite-alginate (HAP-Alg)-reinforced polymeric hydrogel membrane demonstrates remarkable durability, hemocompatibility, and a rapid mineralization capacity, making it a superior alternative to clinically available membranes for GTR [113]. Dubey et al. reported a fiber-reinforced hydrogel that offers unprecedented tunability in terms of its mechanical properties and therapeutic characteristics. This was achieved by integrating a highly porous poly(ε-caprolactone) fibrous mesh, featuring a well-controlled 3D architecture, into bioactive amorphous magnesium phosphate-infused gelatin methacryloyl hydrogels. The fiber-reinforced hydrogel demonstrated positive cellular reactions, notably faster mineralization rates, upregulation of osteogenic-related genes, and enhanced bone formation [114]. Swetha et al. crafted a polymeric hydrogel membrane, reinforced with tricalcium phosphate (TCP)-alginate, aimed at promoting periodontal tissue regeneration. The TCP-reinforced alginate membrane they developed showed hemocompatibility and safety, indicating its appropriateness for use in periodontal therapy as a potent regenerative material [38]. Lee et al. developed an implantable 3D Mo3Se3-IMW-reinforced gelatin-GMA/silk-GMA hydrogel (IMW-GS) to promote osteogenesis and bone formation. The mechanical properties of the 3D-printed IMW-GS hydrogel have been significantly improved, leading to notable progress in the proliferation of human osteoblast cells (HOBs), osteogenic gene expression, collagen deposition, and mineralization [115].

Beyond the regenerative support function of gels, their antimicrobial capacity in periodontitis treatments holds significant importance, with ozonized gels potentially offering valuable assistance [116]. Scribante et al. have evaluated the effectiveness of an ozone-based gel compared to the more prevalent chlorhexidine gels. Their findings revealed that these novel topical treatments not only exhibited antimicrobial properties but also manifested a multitude of other beneficial effects, including demonstrating safe cytocompatibility towards host tissues [117]. Furthermore, Scribante et al. have proposed an antimicrobial gel formulated with postbiotics, lactoferrin, and aloe barbadensis leaf juice powder, suggesting its efficacy for domiciliary treatment of periodontitis [118].

**Table 3:** Summary of biomaterial scaffolds used for periodontal tissue engineering.

Classifications	Definitions		Characteristics	References
Natural biomaterials	Collagen: a natural protein found abundantly in the ECM of various connective tissues		Biocompatibility, biodegradability, hemostatic capabilities, low immunogenicity, mechanical properties	[14,52,53,54]
	Silk fibroin: a natural macromolecular fibrous protein extracted from silk		Biocompatibility, biodegradability, bioactivity, mechanical properties, proteolytic degradability, minimal inflammatory reaction	[55,56,57,58,59]
	Gelatin: a water-soluble protein mixture derived from the partial hydrolysis of collagen		Biocompatibility, biodegradability, promotion of cell growth and adhesion, cytocompatibility, hydrophilicity, water solubility	[60,61,62,63]
	Cellulose: a natural polysaccharide consisting of glucose units linked by beta-1,4-glycosidic bonds		Biocompatibility, biodegradability, mechanical properties, high water retention capacity, high purity and crystallinity, good chemical stability, low cost, ease of processing	[64,65,66,67,68]
	Alginate: a natural polysaccharide found in various species of algae		Biocompatibility, biodegradability, non-toxicity, homogeneity, binding, dispersibility	[69,70,71]
	Chitosan: a natural anionic polysaccharide derived from deacetylated chitin		Biocompatibility, biodegradability, antimicrobial properties, antioxidant activity, hygroscopicity, low antigenicity	[72,73,74,75,76,77]
Synthetic biomaterials	Bioceramics	Calcium phosphates	Biocompatibility, osteoconductivity, slow bioresorbability	[79,80,81,82,83]
		Calcium sulfates	Biodegradability, bioresorbability, osteogenic properties	[84,85]
		Bioactive glasses	Biocompatibility, bioactivity, cytotoxicity, osteoinductive properties, angiogenic properties	[87,88,90]
	Synthetic polymers: high molecular weight compounds consisting of multiple repeating units linked by covalent bonds		Biocompatibility, biodegradability, mechanical properties, wettability, swelling ability, electrostatic effects, hydrolytic degradation, elasticity	[91,92,93,94,95,96,97]
	Metals and alloys	Titanium	Biocompatibility, corrosion resistance, mechanical properties, porosity, osteogenic properties	[98,99,100,101]
		Magnesium	Biocompatibility, mechanical properties	[102,103,104,105]
Hydrogels	Hydrogels: a category of cross-linked three-dimensional hydrophilic polymer networks		Biocompatibility, biodegradability, softness, non-deformability, strong water absorption capacity, high drug utilization rate, high permeability, safety, and convenience	[106,107,108,109,110,111,112,113,114,115]

## 5. Conclusions

The regeneration of periodontal tissue entails a significant degree of intricacy, stemming from the periodontium’s specialized characteristics and hierarchical organization. Given the constraints of conventional treatments for addressing periodontal defects, there is a pressing need for further progression in periodontal regenerative therapy, grounded in tissue engineering principles. The biology of periodontal tissue engineering encompasses an intricate interaction between multiple components such as cells (fibroblasts, osteogenic cells, and immune cells), bioactive molecules (growth factors), and biomaterial scaffolds that mimic the ECM. Among these intricate frameworks, biomaterial scaffolds hold a crucial position in periodontal tissue engineering. They function as a three-dimensional blueprint for cellular growth, fostering proliferation and differentiation, directing tissue regeneration, and serving as vehicles for growth factor delivery. Additionally, their biodegradability and mechanical support are indispensable for achieving successful periodontal tissue regeneration.

Whether it involves the exploration of natural biomaterials or the study of synthetic ones, recent strides in tissue engineering and regenerative medicine across diverse tissue types have ignited promising avenues for periodontal tissue regeneration therapies. Researchers are actively pursuing the development of clinically feasible strategies aimed at regenerating not just a single periodontal tissue type but an entire periodontium, encompassing both soft and hard tissues. This holistic approach holds great potential for restoring tooth function compromised by periodontitis. To realize this vision, multifunctional and multiphasic scaffolds are being designed to mimic the nano-architecture of the ECM, creating an optimal microenvironment for cell proliferation, differentiation, and new tissue formation. Temporal and spatial control of drug/growth factor delivery from these scaffolds is crucial for guiding the precise growth and differentiation of cell types within periodontal defects. Furthermore, bioactive nanoparticles can be integrated into the scaffolds to bolster antioxidative, anti-inflammatory, antibacterial, and angiogenic properties.

The advent of patient-specific, custom-designed 3D scaffolds offers personalized solutions tailored to each individual’s unique needs and characteristics, thereby optimizing the regenerative process and enhancing outcomes. However, it is paramount to underscore that, despite the promising advancements in tissue engineering approaches for periodontal tissue regeneration, their definitive superiority over established treatment modalities has yet to be conclusively demonstrated. This gap primarily stems from a notable scarcity of rigorous in vivo and clinical evaluations within periodontal defect models, which limits our ability to comprehensively assess the efficacy and superiority of these emerging therapies. To mitigate the potential risks of bias and ensure the integrity of periodontal tissue engineering research, the adoption of stringent study designs becomes imperative. These designs must incorporate robust controls that adequately account for confounding factors, randomization to minimize selection bias, and blinded assessments to preserve the objectivity of outcome measures. Such measures not only strengthen the validity of findings but also enhance the credibility and reproducibility of research outcomes. Furthermore, the pursuit of long-term safety and efficacy evaluations represents a cornerstone in the development of safe and effective regenerative strategies. It is crucial to conduct meticulous patient selection, ensuring that individuals enrolled in studies represent the target population and possess the necessary characteristics for a meaningful assessment. Concurrently, compliance monitoring is vital to maintain the integrity of the study protocol and ensure that participants adhere to the prescribed treatment regimen, thereby safeguarding the validity of the research outcomes. Moreover, the identification and exploration of potential challenges and limitations in periodontal tissue engineering research are paramount. These may include, but are not limited to, difficulties in replicating complex tissue architectures, achieving functional integration with host tissues, and addressing the immunological response to implanted biomaterials.

By acknowledging and addressing these challenges, researchers can pave the way for the development of more refined and effective regenerative therapies, ultimately advancing the field towards more definitive conclusions regarding the superiority of tissue engineering approaches. The intensification of research efforts on biomaterial scaffolds, coupled with advancements in fabrication technology, portends a significant expansion in their application for periodontal tissue regeneration in the near future.

## Figures and Tables

**Table 1:** Summary of bioactive molecules and stem cells incorporated in biomaterial scaffolds.

Classifications	Definitions	Roles or Mechanisms
Bioactive molecules	Growth factors	Proteins that stimulate cell proliferation, differentiation, and matrix production, including bone morphogenetic proteins (BMPs), platelet-derived growth factor (PDGF), fibroblast growth factor (FGF), and transforming growth factor-beta (TGF-β)
	Cytokines	Signaling molecules that regulate immune responses, cell migration, and tissue remodeling, such as interleukin-1 (IL-1), interleukin-6 (IL-6), and tumor necrosis factor-alpha (TNF-α)
	ECM components	Natural or synthetic molecules that mimic the composition of the native tissue ECM, such as collagen, fibronectin, laminin, and hyaluronic acid
	Enzymes	Proteins that catalyze biochemical reactions, such as matrix metalloproteinases (MMPs), which can regulate ECM degradation and remodeling during tissue regeneration
	Small molecules and drugs	Compounds that promote angiogenesis, reduce inflammation, or inhibit bacterial growth, such as angiogenic factors like vascular endothelial growth factor (VEGF)
	Genes and gene vectors	Genetic material encoding for growth factors, cytokines, or other therapeutic proteins can be delivered using viral or non-viral vectors to promote tissue regeneration
Stem cells	Fibroblasts	Synthesizing and secreting the ECM components, remodeling the existing tissue to restore its functional and structural integrity, interacting with immune cells and inflammatory mediators to regulate the inflammatory response, secreting a variety of growth factors and cytokines, and responding to mechanical stimuli
	Osteogenic cells	Differentiating into bone-forming cells, participating in the deposition of mineralized matrix and alveolar bone regeneration, reconstituting the periodontal complex, and restoring its functional integrity
	Mesenchymal stem cells (MSCs)	Interacting with the components of the ECM such as collagen, proteoglycans, glycosaminoglycans (GAGs), and several proteins on behalf of variable matrix elasticity and bioactive cues
	Immune cells	Contributing to the clearance of pathogens and damaged tissue, the regulation of inflammatory responses, tissue remodeling, and the maintenance of tissue homeostasis

## Data Availability

Not applicable.
